# Auditory Brainstem Responses to Bone-Conducted Brief Tones in Young Children with Conductive or Sensorineural Hearing Loss

**DOI:** 10.1155/2012/284864

**Published:** 2012-09-04

**Authors:** Jennifer L. Hatton, Renée M. Janssen, David R. Stapells

**Affiliations:** ^1^School of Audiology and Speech Sciences, The University of British Columbia, Vancouver, BC, Canada V6T 1Z3; ^2^Audiology Department, Britsh Columbia's Children' Hospital, Vancouver, BC, Canada V6H 3V4; ^3^British Columbia Early Hearing Program, Provicial Health Services Authority Victoria, BC, Canada V8V 3K3

## Abstract

The bone-conduction (BC) tone ABR has been used clinically for over 20 years. The current study formally evaluated the test performance of the BC tone-evoked ABR in infants with hearing loss. *Method*. By comparing BC-ABR results to follow-up behavioural results, this study addressed two questions: (i) whether the BC tone ABR was successful in differentiating children with conductive versus sensorineural hearing loss (Study A; conductive: 68 ears; SNHL: 129 ears) and (ii) the relationship between BC ABR and behavioural hearing loss severity (Study B: 2000 Hz: 104 ears; 500 Hz: 47 ears). *Results*. Results demonstrate that the “normal” BC-ABR levels accurately differentiated normal versus elevated cochlear sensitivity (accuracy: 98% for 2000 Hz; 98% for 500 Hz). A subset of infants in Study A with elevated BC-ABR (i.e., no response at normal level) had additional testing at higher intensities, which allowed for categorization of the degree of cochlear impairment. Study B results indicate that the BC ABR accurately categorizes the degree of cochlear hearing loss for 2000 Hz (accuracy = 95.2%). A preliminary dBnHL-to-dBHL correction factor of “0 dB” was determined for 2000 Hz BC ABR. *Conclusions*. These findings further support the use of BC tone ABR for diagnostic ABR testing.

## 1. Introduction

Hearing loss is a relatively common sensory impairment that may have a wide variety of harmful effects on the affected child and family if not recognized and managed. Multiple studies have shown that children identified with hearing loss who have treatment and intervention services implemented no later than six months of age perform significantly better on various speech and language measures compared to children identified later (e.g., [1–3]). Thus, early hearing screening and intervention programs, such as the British Columbia Early Hearing Program (BCEHP), have the goal of screening all newborns for hearing loss by one month of age, comprehensive diagnostic assessment by three months of age, and implementation of intervention services and amplification no later than six months of age [4, 5]. 

The tone-evoked auditory brainstem response (ABR) is an important part of the diagnostic test battery and is currently the gold-standard technique for diagnosing type and degree of hearing loss in infants who do not pass hearing screening [5–7]. Several clinical ABR protocol guidelines have been established which recommend and describe the use of the tone-evoked ABR elicited using both air-conducted (AC) and bone-conducted (BC) stimuli in order to characterize the degree and configuration of the hearing loss and to differentiate types (e.g., sensorineural or conductive) of hearing losses [4–6, 8]. Although tone-evoked ABR testing has been used successfully in both research and clinical settings since the 1980s (for reviews, see [9–11]), surprisingly many clinicians today persist in using broadband click stimuli for ABR thresholds, even though the inadequacy of click-ABR threshold has been known and documented for many years (e.g., [12–16]). On a recent (March 22, 2012) search on the World Wide Web, many infant diagnostic ABR protocols were found that continue to use broadband click stimuli to determine ABR thresholds, and published surveys of clinicians' practices show they continue to use clicks (e.g., [17, 18]). 

A review of past and recent literature indicates that the tone-evoked ABR shows good correspondence with subsequent behavioural thresholds for AC stimuli. Correlations between AC tone ABR and behavioural thresholds are typically *r* = .9 or higher for 500 Hz, 2000 Hz, and 4000 Hz (e.g., [19–21]). Across studies, most infants have tone-evoked AC ABR thresholds within 5–10 dB of their pure-tone behavioural thresholds [10]. These results have been confirmed by others as well, and findings have been extended to include results from younger infants [20–25]. 

The most common cause of elevated ABR thresholds in young infants is conductive hearing loss [7, 26]. This is especially so for young infants referred for diagnostic ABR testing after failing one or more newborn hearing screenings. Diagnostic ABR protocols, therefore, must be able to determine whether a significant conductive component is present. When testing older children and adults behaviourally, this assessment is primarily achieved through comparison of air- versus bone-conduction thresholds. For young infants, it is reasonable to expect that the finding of an elevated ABR threshold to air-conduction stimuli with ABR present to bone-conduction stimuli at normal levels would indicate the presence and degree of a conductive hearing loss; if ABR thresholds to bone-conduction stimuli are elevated, a sensorineural component is present [15, 16]. As with air-conduction testing, bone-conduction ABR testing must use frequency specific rather than broadband stimuli (i.e., brief tones rather than clicks) [4–8, 11]. Current comprehensive diagnostic protocols for infants (e.g., [4, 8]) emphasize the importance of obtaining bone-conduction information early in the ABR test sequence (i.e., as soon as an elevation in air-conduction thresholds is indicated)—this information is needed to determine the next test step and is important both for appropriate followup and for parent counselling. Unfortunately, many clinicians routinely fail to obtain ABR results for bone-conduction stimuli after finding elevated air-conduction threshold(s), relying instead on acoustic immittance results. However, a flat tympanogram cannot quantify the amount of conductive hearing loss and does not preclude SNHL; BC results are required.

Tone ABRs to bone-conducted stimuli also have a long history of research and clinical use, dating back at least 20 years [27–30]. The current Joint Committee on Infant Hearing (JCIH) [5] guidelines emphasize the importance of BC-ABR testing for distinguishing between conductive and sensorineural hearing loss and tone-evoked BC-ABR testing is routinely used in large programs, such as the BCEHP [4] and the Ontario Infant Hearing Program (OIHP) [8]. Despite its clinical use and importance for clinical assessment, surprisingly few published data exist in the peer-reviewed literature regarding BC tone ABR testing in infants [20, 29–33]. Additionally, the majority of these published data pertain to infants having normal hearing or conductive hearing loss; very few data regarding BC-ABR testing in infants with sensorineural hearing loss have been published.

The first study of the tone ABR to BC tones in infants was carried out by Stapells and Ruben [29], who investigated the tone ABR evoked by 500 Hz and 2000 Hz BC brief tones in 48 infants (mean age: 6.1 months) with either normal hearing (24 ears) or conductive hearing loss (41 ears). Their results demonstrated that the majority (94–100%) of infants with normal cochlear function exhibit ABRs to BC stimuli at 30 dBnHL for 2000 Hz and at 20 dBnHL for 500 Hz. These levels were suggested as the “normal” levels for clinical testing, separating infants with likely normal cochlear thresholds from those with likely sensorineural hearing loss [15, 16, 34]. These BC levels are currently used by many clinicians, including those involved with the BCEHP and the OIHP [4, 8]. More recently, Vander Werff and colleagues [20] assessed BC-ABR thresholds in a group of infants with normal hearing (500 Hz: *N* = 40; 2000 Hz: *N* = 40). Vander Werff and colleagues did not provide results which would allow determination of “normal” levels (i.e., cumulative response presence); however, mean BC tone ABR thresholds for the normal infants reported by Vander Werff and colleagues were similar (i.e., within 5–7 dB, in dB re: 1 *μ*N) to those reported for normal infants by Stapells and colleagues [29, 30]. In addition to the above studies, two other studies have investigated BC tone-ABR thresholds in normal hearing infants. Foxe and Stapells [30] studied 500 and 2000 Hz BC-ABR responses in a small sample (8-9 infants; mean age 4.8 months) of normal hearing infants. Cone-Wesson and Ramirez [32] investigated BC-ABR thresholds at 500 Hz and 4000 Hz, but not at 2000 Hz, in a group of very young infants (*N* = 60; aged 1-2 days) at low risk for hearing loss. Notably, the 500 Hz BC-ABR thresholds of their very young infants were significantly lower (better) than those reported for older infants [20, 29, 30], possibly related to maturation. It is difficult to compare these results due to the large age differences, as well as the lack of BC 2000 Hz data. A third study used a high-pass noise masking paradigm and demonstrated that infants' BC tone ABRs show reasonable cochlear place specificity, similar to that of adults [33]. Currently, there are too few infant ABR data for 1000 and 4000 Hz BC tones to suggest appropriate normal levels for these frequencies; thus, current BCEHP and OIHP infant ABR protocols do not test these frequencies using bone-conducted stimuli [4, 8, 11]. 

Two studies have investigated the BC tone-ABR in groups of infants with conductive loss. Stapells and Ruben [29] recorded the BC tone-ABR in infants with otitis media (25 ears) or external auditory canal atresia (16 ears). Vander Werff and colleagues [20] looked at a group of infants with conductive hearing loss (500 Hz: 23 ears; 2000 Hz: 9 ears). Stapells and Ruben [29] demonstrated no differences in BC-ABR detectability at 500 and 2000 Hz in groups of infants with normal hearing and conductive hearing loss. Along the same line, Vander Werff and colleagues [20] showed that mean BC-ABR thresholds and latency-intensity functions were similar between infants with normal hearing and conductive hearing loss, suggestive of similar cochlear processing. 

Very few published data exist regarding the BC tone ABR in infants with sensorineural hearing loss. Based on anecdotal case studies (e.g., [10, 11, 27, 35]) and many years of clinical experience [4, 8, 10], the BC tone ABR appears to accurately predict type of hearing loss. However, the only formal study is that of Vander Werff and colleagues [20], who obtained results from a very small group of infants with sensorineural hearing loss (500 Hz: *N* = 2; 2000 Hz: *N* = 9). Furthermore, these researchers presented only ABR air-bone gap results and did not present any mean (or individual) BC-ABR thresholds. Results were not analyzed in such a way as to allow determination of BC-ABR test performance. 

As described above, the published BC tone-ABR literature, especially for infants with sensorineural hearing loss, is surprisingly lacking despite the many years of clinical experience. Importantly, to date, no study has evaluated the test performance of the tone-evoked BC-ABR in infants. To address this, the current study compared BC tone-ABR results, obtained as part of a diagnostic ABR, with followup behavioural results for 108 infants with conductive or sensorineural hearing loss, with the goal of (i) investigating whether BC-ABR was successful in differentiating children with conductive versus sensorineural hearing loss (test performance) and (ii) determining the relationship between BC-ABR and the subsequent severity of the behavioural cochlear hearing loss, if any.

## 2. Methods

### 2.1. Participants

Individuals with results included in this study were 108 young children referred to the British Columbia's Children's Hospital (BCCH) Audiology Department. The referrals for diagnostic ABR assessments were from both internal (i.e., within BCCH) and external (community) sources. Children presented with a variety of risk factors (e.g., referral from hearing screening (note that BCCH is not one of the designated diagnostic sites for referrals from hearing screening in British Columbia. A small number of infants from screening are seen at BCCH in special circumstances, such as those who are very ill or who live in remote regions of the province), ototoxicity, delayed speech and language, neonatal insult, and/or other concomitant medical issues). The average age (corrected for prematurity) at the time of the initial diagnostic ABR assessment was 20.9 months (SD = 16.6; median = 18.7); the average amount of time between the ABR assessment and subsequent behavioural assessment was 13.3 months (SD = 14.9; median = 9.8). 

The chart review examined diagnostic ABR assessments that occurred between 2005 and 2011 and was approved by the Clinical Research Ethics Board of the University of British Columbia. The time period was chosen as it followed the introduction of a new tone-ABR protocol in the Audiology Department [4]. In general, assessments were included in the study if (i) BC-ABR testing had been completed at 2000 Hz and/or 500 Hz, and (ii) reliable behavioural results had been obtained for the child subsequent to the ABR assessment. As a result of requiring BC-ABR testing for inclusion, all ABR assessments included in this study involved abnormal AC ABR thresholds (hearing thresholds ≥30 dB estimated behavioural hearing level (eHL)) for at least one of 500 and 2000 Hz, as these are the two frequencies at which BC-ABR recordings are obtained (as per the BCCH/BCEHP ABR protocol, BC recordings are presently only obtained for 500 and 2000 Hz and only if AC thresholds are elevated. BC recordings for other frequencies are not obtained as normative data and criterion values for “normal” levels have not been established [4]). If multiple behavioural test results were obtained for a given subject, the behavioural test closest in time to ABR testing and/or with most complete results and good reliability was chosen. 

The chart review indicated that 558 children had undergone diagnostic ABR assessments during the period of this review. Approximately half (46%) of the children in the initial chart review were excluded because they had normal ABR results and therefore no BC-ABR results. Additional reasons for exclusion were (i) no reliable behavioural results were available, (ii) the ABR indicated a unilateral loss (i.e., normal hearing for one ear) and subsequent behavioural testing was only obtained in soundfield, (iii) the hearing loss was clearly progressive, (iv) the child had evidence of auditory neuropathy spectrum disorder, or (v) BC-ABR results were inconclusive. After exclusion, results for 108 infants were included. Not all infants had hearing loss in both ears and/or had complete results (i.e., both ears with both 500 and 2000 Hz results); therefore, the study includes results for a maximum of 138 ears at 2000 Hz and 59 ears at 500 Hz.

### 2.2. Sedation

 Generally, patients seen for ABR assessments aged over six months were sedated to ensure sufficient sleep time (87/108 participants). The sedative, when utilized, was chloral hydrate, prescribed by the patients' otolaryngologist, and administered and monitored by the sedation clinic nurse, similar to the procedure described in the American Academy of Pediatrics Guidelines [36, 37]. 

### 2.3. ABR Parameters

All assessments were carried out in a double-walled, sound-attenuated booth using the Intelligent Hearing Systems (IHS) SmartEP system. The ABR was assessed to air conduction at 500, 1000, 2000, and 4000 Hz; bone conduction at 500 and 2000 Hz, where indicated, and to high-intensity clicks, where indicated. Details of the BCCH/BCEHP ABR protocols and parameters are available on the web [4] and in the literature [11]. Briefly, AC-ABR stimuli were presented using insert earphones (Etymotic ER-3A) and BC-ABR stimuli were presented via a Radioear B-71 bone vibrator. Stimuli were brief tones (5-cycle duration, exact-Blackman window) presented at a rate of 39.1/second. AC and BC stimuli were calibrated in dBnHL using 0 dBnHL calibrations provided by Stapells [11] (normal hearing levels (nHL) for AC- and BC-ABR stimuli are referenced to adult behavioural thresholds, as are hearing threshold levels (HL) for paediatric behavioural testing). ABRs were recorded with the noninverting electrode placed on the high forehead (Fpz) and inverting electrodes on the ipsilateral and, for BC ABR, contralateral mastoids. An electrode placed on the forehead served as ground. This setup allowed the simultaneous recording of two EEG channels ((i) high forehead to left mastoid (Fpz-M2), and (ii) high forehead to right mastoid (Fpz-M1)) which were obtained for all BC-ABR recordings (as per the BCCH/BCEHP ABR protocol, comparison of the ipsilateral (i.e., side where stimulus transducer is placed) and contralateral EEG channel ABR recordings is used to determine responding ear. Normally, a larger or earlier wave V in the ipsilateral EEG channel indicates the responding cochlea; however, a larger or earlier response in the contralateral EEG channel is abnormal and suggests the contralateral ear is producing the response [11, 29]. In the current study, for 101 infants, results in the contralateral EEG channel did not change BC-ABR interpretation (i.e., responses were either absent in both channels or the ipsilateral response was clearly better). In only seven infants was the interpretation changed by a larger or earlier response in the contralateral channel). Interelectrode impedances were less than 3 kOhms at 30 Hz. The EEG was amplified and filtered using a bandpass of 30–1500 Hz. Trials containing amplitudes greater than ±25 *μ*V were automatically rejected. Averaging was carried out using a 24.6 ms poststimulus analysis time. At least two to three replications of 2000 trials each was always obtained for threshold bracketing conditions.

### 2.4. Diagnostic ABR Protocol

The BCCH/BCEHP tone-ABR protocol test sequence emphasizes efficiency and obtaining information in a prioritized fashion. A detailed description of this diagnostic ABR protocol is freely available on the World Wide Web [4]. Key features of the protocol are (1) to commence testing at a low (target level for “normal”) intensity (“Normal” dBnHL values are as follows: AC 500 ≤ 35, AC 1000 ≤ 35, AC 2000 ≤ 30, AC 4000 ≤ 25, BC 500 ≤ 20, and BC 2000 ≤ 30); (2) to switch ears immediately and frequently; (3) to obtain recordings to BC stimuli as soon as it has been determined that an AC response is elevated; (4) to initially use a relatively large step size (i.e., greater than 10 dB) that allows for a quick bracketing of thresholds, when elevated, and end with a maximum step size of 10 dB. If a clear reproducible response is obtained to AC recordings at the target “normal” intensity, that ear/frequency is said to be “normal,” and threshold below this normal intensity is not obtained. If AC responses are not present at the normal intensity (i.e., elevated AC), BC recordings are obtained to determine whether an elevated AC threshold is conductive or sensorineural in nature; however, it is not used to estimate the size of the air-bone gap, if present. Until recently [4], actual BC-ABR thresholds were not routinely pursued; rather, testing was often only done at the normal BC intensity and, if no response was present, then at the maximum BC intensity (60 dBnHL for 2000 Hz; 50 dBnHL for 500 Hz). Testing is initiated at AC 2000 Hz (both ears sequentially), and proceeds directly to BC 2000 Hz for those ear(s) for which AC 2000 Hz is elevated. 500 Hz (AC and BC) is given second priority after 2000 Hz.

### 2.5. Classification

#### 2.5.1. ABR-Based Sensorineural versus Conductive Hearing Loss Categorization

ABR-based categorization was carried out and analyzed for each frequency (2000 and 500 Hz) independently. ABR results were included in the sensorineural group for each frequency if AC- and BC-ABR results were elevated at that frequency. ABR results were included in the conductive group for each frequency if AC ABR results were elevated and BC-ABR results were normal at that frequency.

#### 2.5.2. Behavioural Followup Classification Scheme

 BC-ABR results were compared to behavioural results. However, not all children had BC testing completed at the time of behavioural followup; thus, behavioural results were divided into two subgroups, depending on whether or not BC behavioural results were available for those participants with AC elevations. Participants with “certain” hearing status had either normal AC behavioural thresholds on followup (e.g., resolution of a transient conductive hearing loss) or had followup BC behavioural results available (2000 Hz: 82 ears; 500 Hz: 50 ears). However, not all the participants satisfied the criteria for “certain” hearing status. For these additional children, the combination of elevated AC behavioural thresholds and normal acoustic immittance results allowed us to infer elevated cochlear hearing status. These children without followup BC behavioural results were included in the “presumed SNHL” group (2000 Hz: 56 ears; 500 Hz: 9 ears), thereby increasing the overall sample size of the SNHL group (data for ears with abnormal middle-ear status and no BC results at the time of behavioural assessment were excluded because type of hearing loss could not be determined).

### 2.6. Study A: Test Performance: Accuracy of BC-ABR in Evaluating Type of Hearing Loss (SNHL versus CHL)

 This study first evaluated the accuracy of ABR BC recordings in correctly characterizing type of hearing loss in 108 infants with hearing loss. Participants were categorized as having hearing loss of either sensorineural (*N* = 129 ears) or conductive (*N* = 68 ears) type, based on the results of the ABR assessment. The number of infants who were correctly identified as per subsequent behavioural testing as having either CHL or SNHL was then determined.

### 2.7. Study B: BC-ABR Severity and Degree of Hearing Loss: Relationship between BC-ABR Results and AC Behavioural Thresholds

 Study B evaluated the relationship between BC-ABR and AC behavioural results. At the time of this study, the BCEHP protocol did not mandate that BC-ABR thresholds be obtained; therefore, only a subset of the infants included in the first part of this study had BC-ABR results completed at multiple intensities. Results of a total of 85 infants were included in Study B. Seventy infants (104 ears) had 2000 Hz BC-ABR thresholds that fell within the following three categories: (i) ≤30 dBnHL (i.e., normal; 29 ears); (ii) 35–60 dBnHL (elevated; 26 ears); (iii) >60 dBnHL (no response at maximum intensity; 49 ears). Twenty-one ears had actual BC-ABR thresholds obtained at 2000 Hz. Thirty-eight infants (47 ears) had 500 Hz BC-ABR thresholds that fell within the following three categories: (i) ≤20 dBnHL (i.e., normal; 37 ears), (ii) 25–50 dBnHL (i.e., elevated; 6 ears), and (iii) >50 dBnHL (no response at maximum intensity; 4 ears). Six ears had actual BC-ABR thresholds obtained at 500 Hz. Study B compared the correspondence between these three BC-ABR categories with behavioural outcomes. For the small number of infants for which actual BC-ABR thresholds were obtained, threshold difference scores (i.e., BC-ABR minus AC or BC behavioural) were calculated. 

## 3. Results

### 3.1. Study A

**Figure 1:**
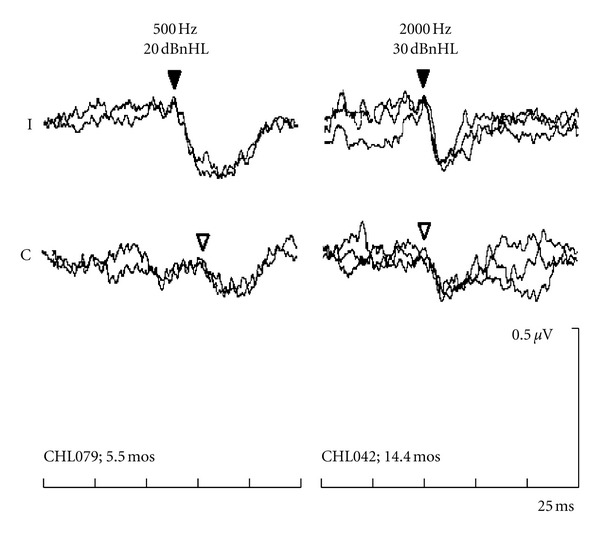
ABRs to 500 and 2000 Hz BC tones, presented at 20 and 30 dBnHL respectively, recorded from two infants with conductive hearing loss at the time of ABR testing. Age at ABR testing indicated on the bottom. These infants had normal hearing to air-conduction stimuli at behavioural followup. “I” equals the ipsilateral channel (EEG channel ipsilateral to the BC transducer). “C” equals the contralateral channel (EEG channel contralateral to the BC transducer). Waves V are indicated by the triangles (filled = ipsilateral; open = contralateral).

This study evaluated the accuracy of BC-ABR recordings to correctly identify conductive versus sensorineural hearing loss.  Figure 1 shows 500 and 2000 Hz BC-ABR results present at the normal BCEHP levels recorded from two infants—one infant at 500 Hz (left); the other infant at 2000 Hz (right)—both with elevated AC ABR results (i.e., conductive hearing loss). Typical for infants with normal cochlear sensitivity, these infants' waves V in the ipsilateral EEG channels are earlier and larger than those seen in the contralateral EEG channels [16, 29]. Behavioural testing was consistent with normal hearing. 

**Figure 2:**
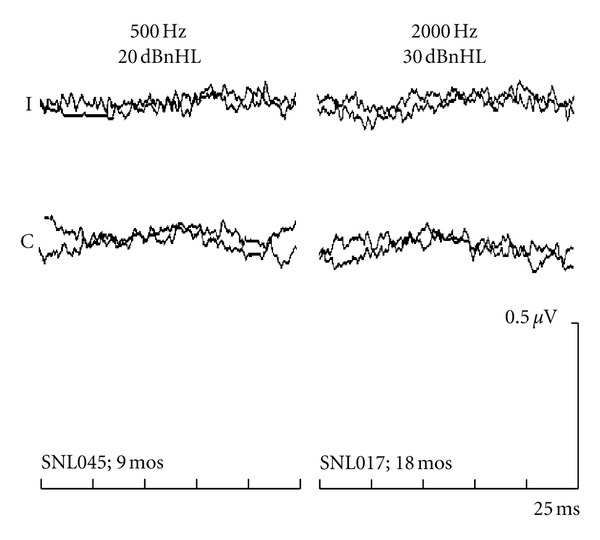
“Elevated” ABR results (i.e., no response at normal intensities) for 500- and 2000-Hz BC tones recorded from two infants with sensorineural hearing loss. Age at ABR testing indicated on the bottom. On behavioural follow up, both infants had sensorineural hearing loss.

Figure 2 demonstrates “elevated” BC-ABR results (i.e., no response at the normal intensities) to 500 and 2000 Hz bone-conduction tones recorded from two infants with elevated AC- and BC-ABR results (i.e., sensorineural hearing loss). No clear wave V is detected at the normal BC-ABR levels. For the infant with 500 Hz results, behavioural testing at age 24 months indicated a profound sensorineural hearing loss. For the infant with 2000 Hz results, behavioural testing at 41 months was consistent with moderate sensorineural hearing loss.

The examples in Figures 1 and 2 are typical of the results obtained from the group of infants in Study A.  Table 1 compares BC-ABR determination of cochlear status with actual cochlear status as determined by behavioural followup. The BC-ABR results accurately categorized cochlear status, erring in only 2.0% (4/197) of the comparisons (across frequencies), with similar results overall for the two frequencies. Results were equally accurate when only the “certain” data were considered. Of the four errors, three had mild sensorineural hearing loss (30–40 dBHL) and one had moderate sensorineural hearing loss on followup that was missed by BC ABR. Overall, there were 13 comparisons with mild sensorineural hearing loss in Study A; the ABR missed only three of these mild losses.

Results of Study A demonstrate, for a large number of infants, that the BC-ABR normal levels of 30 dBnHL for 2000 Hz and 20 dBnHL for 500 Hz accurately differentiate normal versus elevated cochlear sensitivity in infants with conductive or sensorineural hearing loss. Across both frequencies, sensitivity was 97%; specificity was 100%. These results are consistent with clinical experience and corroborate clinical ABR protocols used by the BCEHP [4] and other programs (e.g., [8, 16]).

### 3.2. Study B

 Although the results of Study A showed that BC-ABR is accurate in identifying the type of hearing loss, because these BC data were only obtained at the “normal” levels, Study A was not able to provide information regarding the degree of cochlear impairment. However, a subset of infants with elevated BC-ABR results in Study A were also tested at higher BC intensities, usually at the maximum allowable BC-ABR intensities (i.e., 60 dBnHL for 2000 Hz and 50 dBnHL for 500 Hz). By comparing these results to behavioural followup, this allowed us to assess how well BC-ABR categorized the severity of cochlear impairment. 

**Figure 3:**
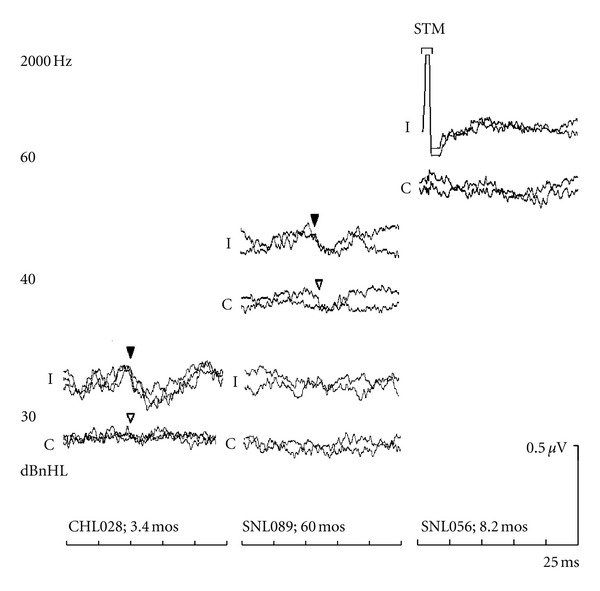
Left panel: ABRs to 30 dBnHL BC 2000 Hz tones recorded from an infant with conductive hearing loss at the time of ABR testing, who had normal hearing at behavioural followup. Middle Panel: “Elevated” 2000 Hz BC-ABR results (threshold = 40 dBnHL) from a child who showed a moderate sensorineural hearing loss at behavioural followup. Right panel: “Elevated” 2000 Hz BC-ABR results (threshold > 60 dBnHL) from an infant who showed profound sensorineural hearing loss behaviourally. STM indicates the region of uncancelled stimulus artifact.

Figure 3 presents results for 2000 Hz from three young children, typical of the group results, where BC-ABR was used to categorize cochlear sensitivity into three categories: (i) normal, (ii) 35–60 dBnHL, and (iii) >60 dBnHL. Results for the 3.4-month-old infant on the left panel indicate normal cochlear sensitivity, which was confirmed at behavioural followup at 32 months of age. The middle panel shows results for a 5-year-old indicating elevated, but present at 40 dBnHL, BC-ABR results; behavioural followup was consistent with a moderate SNHL. The right panel displays BC-ABR results from an infant with profound SNHL (determined by behavioural followup) showing absent BC-ABR at the maximum level of 60 dBnHL.

**Figure 4:**
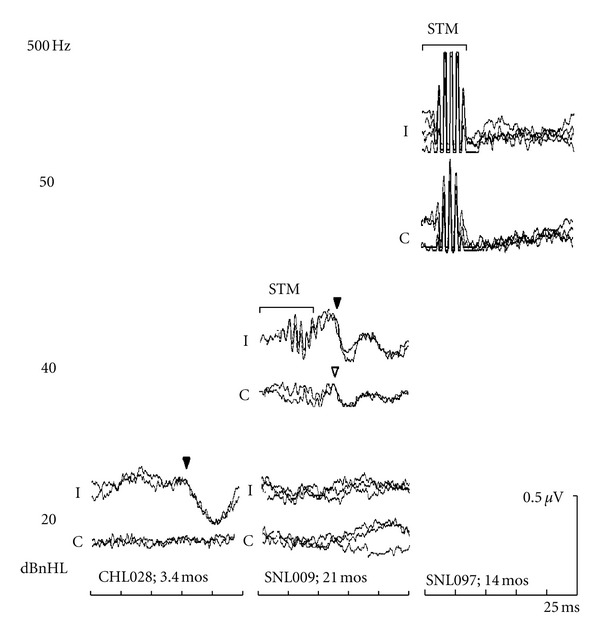
Left panel: ABRs to 20 dBnHL BC 500 Hz tones recorded from an infant with conductive hearing loss at the time of ABR testing, who showed normal hearing at behavioural followup. Middle panel: “Elevated” 500 Hz BC-ABR results (threshold = 30–40 dBnHL) from an infant who showed a moderate sensorineural hearing loss behaviourally. Right panel: “Elevated” 500 Hz BC-ABR results (threshold > 50 dBnHL) from an infant with a severe/profound sensorineural hearing loss bilaterally at followup. STM indicates the region of uncancelled stimulus artifact.

Figure 4 displays typical results for 500 Hz from three young children. Similar to the 2000 Hz results described above, BC-ABR was used to categorize cochlear sensitivity into three categories: (i) normal, (ii) 25–45 dBnHL, and (iii) >50 dBnHL. The left panel shows results from a 3.4-month-old infant with normal cochlear sensitivity; behavioural followup revealed normal hearing. The middle panel shows results for a 21-month-old infant indicating elevated BC-ABR results, but a clear response present at 40 dBnHL; behavioural followup was consistent with a moderate SNHL. Results for the 14-month-old infant with profound SNHL on the right panel show absent BC-ABR at the maximum level of 50 dBnHL. 

The above examples in Figures 3 and 4 indicate that the BC-ABR was able to appropriately categorize degree of cochlear impairment as determined by behavioural testing. In order to assess results for the group data, two comparisons were made between BC-ABR and behavioural outcome results. First, we compared BC-ABR to behavioural AC thresholds to maximize the number of data points (there were many instances where behavioural BC thresholds were not available); AC behavioural results were only used when there was evidence of no conductive overlay. In cases of permanent conductive hearing loss, where behavioural AC thresholds were not reflective of cochlear sensitivity, behavioural BC results were used in place of AC thresholds. In a subsequent analysis, we focused specifically on comparisons between BC-ABR and BC behavioural, which resulted in a smaller sample size for these latter analyses. 

**Figure 5:**
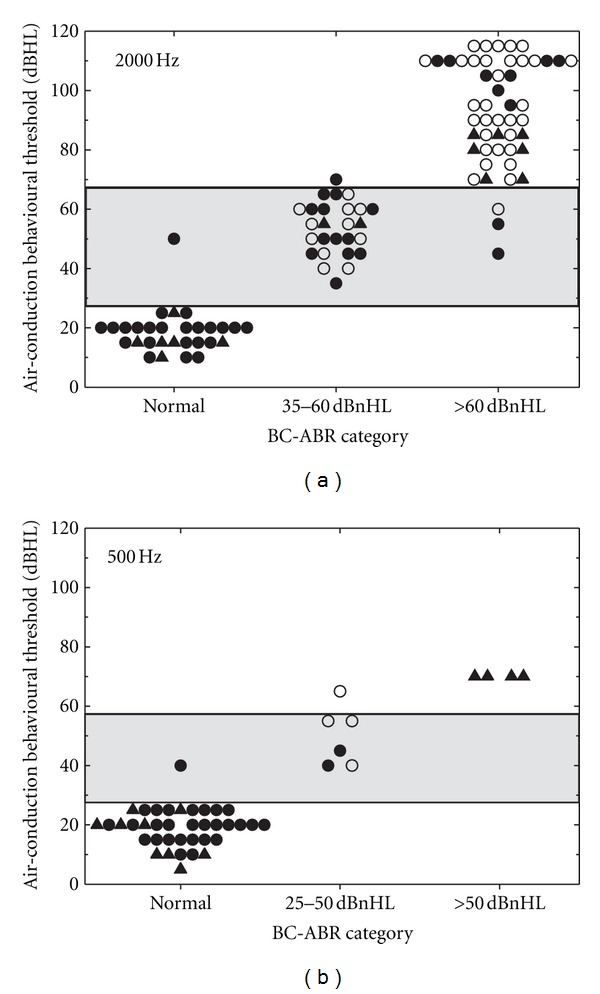
Comparison of BC-ABR threshold category to AC behavioural threshold data for 2000 Hz (a) and 500 Hz (b). Filled circles indicate ears with “certain” hearing status, whereas open circles indicate ears with “presumed SNHL.” Filled triangles represent ears where BC behavioural results were used (i.e., when AC behavioural results did not reflect cochlear status). Behavioural categories are indicated by horizontal lines/shading. See text for details.

Figure 5 shows the results of the first comparison of BC-ABR category to (primarily) AC behavioural threshold data for 2000 Hz (Figure 5(a); 104 ears) and 500 Hz (Figure 5(b); 47 ears). With only a few exceptions, as BC-ABR category increases in severity so do AC behavioural thresholds. Although present for both frequencies, there are fewer data for 500 Hz in the more severe hearing loss categories, and therefore conclusions are limited for this frequency. 

Based on the results of Figure 5, we divided the behavioural results into three categories of severity of hearing impairment and then quantified how well the BC-ABR categories corresponded to these three behavioural threshold categories.  Table 2 shows the results of these comparisons. For 2000 Hz, where the most data are available, the Spearman rank correlation coefficient was .96; BC-ABR correctly determined behavioural hearing loss category in 95.2% of the comparisons. For 500 Hz, the Spearman rank correlation coefficient was .95; BC-ABR correctly determined behavioural hearing loss category in 95.7% of the comparisons. As mentioned previously, the data for 500 Hz are limited due to the small number of participants with hearing loss in this analysis with BC-ABR results above the normal level.

**Figure 6:**
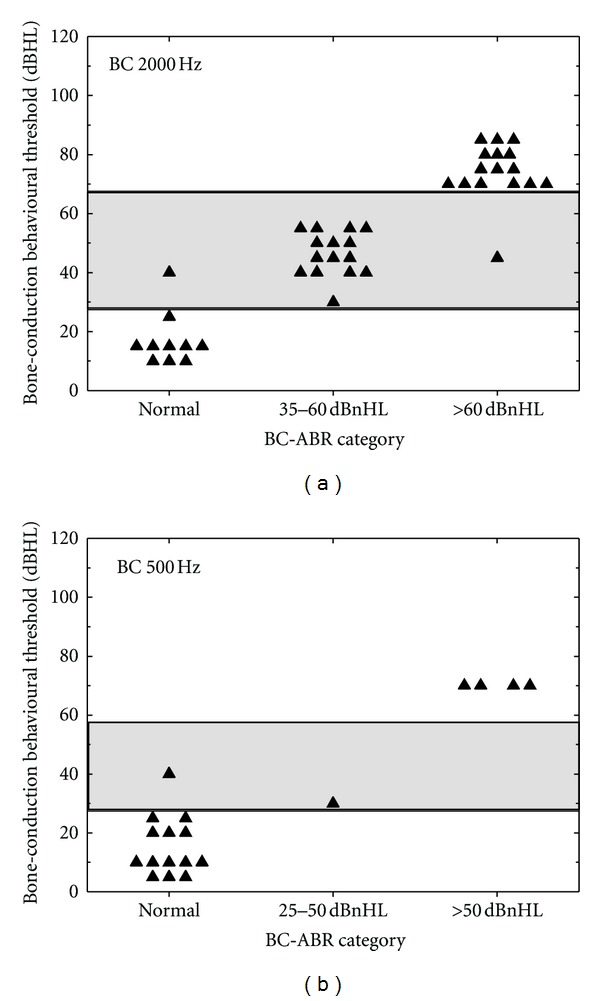
Comparison of BC-ABR threshold category to BC behavioural threshold data for 2000 Hz (a) and 500 Hz (b). Behavioural categories are indicated by horizontal lines/shading.

Figure 6 shows the results of the second comparison where we compared the BC-ABR only to behavioural BC threshold data for 2000 Hz (Figure 6(a); 41 ears) and 500 Hz (Figure 6(b); 19 ears). Because of the more restrictive inclusion criteria, sample's sizes are smaller, especially at 500 Hz. Similar to the results shown in Figure 5, 2000 Hz results show that as BC-ABR hearing loss severity category increases so does BC behavioural threshold. We do not see the large discrepancies between BC-ABR and BC behavioural as we did when comparing BC-ABR to AC behavioural. For 500 Hz, there is a suggestion of a correspondence between BC-ABR category and BC behavioural thresholds, but due to limited data with hearing loss, results are inconclusive.

Table 3 shows the results of the comparisons in Figure 6. For 2000 Hz, the Spearman rank correlation coefficient was .96; BC-ABR correctly determined behavioural hearing loss category in 92.8% of the comparisons. For 500 Hz, the Spearman rank correlation coefficient was .93; BC-ABR correctly determined behavioural hearing loss category in 94.7% of the comparisons. As noted above, due to limited data for hearing loss, the results of this analysis are inconclusive for 500 Hz. 

The preceding analysis was unable to provide ABR-minus-behavioural threshold difference scores for the purpose of determining correction factors because they included nonthreshold data (e.g., response present at the normal levels indicating normal cochlear sensitivity; no response at the maximum intensity levels). Nevertheless, there was a subset of cases in the middle BC-ABR category where actual BC-ABR thresholds were obtained and thus difference scores could be calculated. For 2000 Hz, 21 ears had both BC-ABR and AC behavioural thresholds, with the BC-ABR on average better than behavioural thresholds (mean = −6.2 dB; SD = 8.9; *t* = −3.2, *df* = 20, *P* < .005). Most (71%) of these comparisons were within 10 dB, 91% were within 15 dB, and all were within 25 dB. Only 2 ears (9.5%) showed BC-ABR thresholds worse than AC behavioural thresholds. 

Twelve ears had both ABR and behavioural BC thresholds at 2000 Hz, with the BC ABR on average being slightly but not significantly better (mean = −1.3 dB; SD = 8.3; *t* = −0.5, *df* = 11, *P* = .61). Most (83.3%) of these comparisons were within 5 dB, 92% were within 15 dB, and all were within 20 dB. Again, BC ABR was better than or equal to behavioural BC thresholds in most cases. Only 2 ears (17%) showed BC-ABR thresholds worse than BC behavioural thresholds. 

Threshold difference scores for 500 Hz were available for only 6 ears, and only for BC-ABR compared to AC behavioural. BC-ABR threshold for 500 Hz was always better than AC behavioural thresholds (mean = −16.7; SD = 10.8; *t* = −3.8; *df* = 5, *P* = .013). All difference scores were within 30 dB.

Results of Study B indicate that BC-ABR accurately categorizes the degree of cochlear hearing loss at 2000 and 500 Hz. Further, when 2000 Hz BC-ABR thresholds are elevated (i.e., >30 dBnHL at 2000 Hz) and actual BC-ABR thresholds are obtained, the BC-ABR thresholds are usually within 5 dB of the behavioural BC thresholds. Because few actual BC-ABR thresholds were obtained for 500 Hz, conclusions are limited.

## 4. Discussion

The present studies show that the BC tone ABR accurately differentiates normal versus elevated cochlear sensitivity in infants with conductive or sensorineural hearing loss, both for 500 Hz and 2000 Hz (Study A). Additionally, at least for 2000 Hz, the degree of cochlear impairment can be accurately categorized (Study B). Finally, the present studies also determined difference scores which might be used to predict behavioural thresholds when elevated BC tone-ABR thresholds are obtained (Study B). These studies are the first to formally assess the performance (e.g., accuracy of categorization of hearing loss type, as well as degree of bone-conduction elevation) of the BC tone-evoked ABR in groups of infants with conductive or sensorineural hearing loss. Prior to this, a long history of clinical use together with studies assessing infants with conductive hearing loss, and a few anecdotal or small-N studies with sensorineural hearing loss were relied on to support the use of BC tone ABR for clinical practice.

### 4.1. Study A

As shown in Study A, nearly all infants showing responses to BC tones at 30 dBnHL at 2000 Hz (28/31 ears) and/or 20 dBnHL at 500 Hz (36/37 ears) had normal cochlear sensitivity at these frequencies on followup. Similarly, all those infants with no response at these intensities had sensorineural hearing impairment (2000 Hz: 107/107 ears; 500 Hz: 22/22 ears) on followup. Ears with mild sensorineural hearing loss (30–40 dBHL), for the most part (10/13 ears), showed elevated BC ABR. These results support the recommendations made by Stapells and colleagues to use these BC intensities to separate normal versus elevated cochlear sensitivity in infants [10, 11, 15, 16, 29] and they are currently used by the BCEHP [4] and OIHP [8]. 

### 4.2. Study B

When bone-conduction responses are elevated (i.e., not present at the normal levels), it is useful to determine the degree of sensorineural elevation by assessing higher intensities. Until recently, the BCEHP protocol only required determination of “normal” versus “elevated” BC ABR, with threshold searching optional after completing other mandatory elements (the recently revised BCEHP protocol now requires determination of BC-ABR thresholds at 2000 Hz (and at 500 Hz if the only AC elevation is at 500 Hz) [4]). In practice, clinicians in the current study often tested the maximum BC level when no response was present at the normal level. In fewer cases, actual BC-ABR thresholds were obtained, typically at 2000 Hz. Thus, for this study, for a reasonably large group of infants at 2000 Hz (29 ears with normal cochlear sensitivity; 75 ears with sensorineural hearing loss), we were able to determine that the BC-ABR accurately estimated (92.8% accuracy) cochlear status into three categories (i.e., normal; 30–65 dBHL; >65 dBHL). Similar accuracy was found for 500 Hz (94.7%); however, interpretation for 500 Hz is limited due to the small number of ears with sensorineural hearing loss (37 ears with normal cochlear sensitivity; 10 ears with sensorineural hearing loss) that we were able to classify into the three categories at this frequency. The above results include both “certain” and “presumed” sensorineural hearing loss. Similar results were obtained when only “certain” cases were considered, although with about half the number of ears. 

 Ideally, one wants to know the correction factor that allows one to convert BC-ABR thresholds (in dBnHL) to estimated behavioural BC thresholds (in dBeHL). To do this, one needs actual BC-ABR thresholds. As noted previously, the earlier BCEHP protocol did not mandate BC-ABR thresholds, thus, actual thresholds were not obtained for most of the infants in this study. Additionally, actual BC-ABR thresholds were not available for the ears with normal cochlear sensitivity as testing was only carried out at the normal BC-ABR level. Further, many infants with significant sensorineural hearing loss had no response at the maximum level, and thus thresholds could not be determined. Therefore, when available, threshold data were only available for those infants whose hearing loss fell in the middle BC-ABR category. Actual threshold results for 2000 Hz were available for 21 ears that had both BC-ABR and AC behavioural thresholds and 12 ears that had both BC-ABR and BC behavioural thresholds. The BC-ABR was, on average, essentially the same as the BC behavioural (BC ABR was 1.3 dB better than BC behavioural) and 6.2 dB better than the AC behavioural. Based on these results, a preliminary correction factor to estimate BC behavioural thresholds for 2000 Hz from the BC ABR would be 0 dB (i.e., 40 dBnHL BC ABR = 40 dBeHL BC behavioural); this is similar to published ABR-minus-behavioural difference scores for air-conduction ABR at 2000 Hz [11, 20]. This is also consistent with little or no maturation of mid- to high-frequency BC thresholds, with infant thresholds within 5 dB of adult thresholds above 1000 Hz [20, 30, 38]. The current data cannot provide a reliable correction factor for 500 Hz BC ABR. It is likely to be a larger correction (e.g., BC-ABR is 10–20 dB better than behavioural followup) as many studies have shown low-frequency BC thresholds to be significantly better in infants than in adults [20, 30, 32, 38].

### 4.3. Outliers

As noted in the results, in a few cases (4/197 ears), the BC-ABR categorization of normal versus elevated cochlear sensitivity differed from that of behavioural followup categorization. In one case, behavioural followup showed a moderate cochlear hearing loss, whereas the BC-ABR was normal and it is possible that the behavioural thresholds did not reflect true sensitivity (e.g., although behavioural results were considered to have “good” reliability, this child had a significant mental handicap). In the other three cases, behavioural followup showed mildly elevated thresholds compared to the normal BC ABR. There are several possible explanations. First, it is possible that the normal levels miss mild (30–40 dBHL) cochlear hearing loss. However, of the 13 ears with mild cochlear hearing loss in Study A, only 3 were missed by BC ABR. Equally likely, progressive and/or late onset hearing loss can also explain these results as there were 5, 10, and 16 months between ABR and followup behavioural testing for these three children. Indeed, previous studies have shown that 5–30% of children with sensorineural hearing loss show progression in their thresholds (for review see: [39]), so it would be expected that a small number of children in our study would show worse thresholds on followup. 

### 4.4. Limitations of This Study

As a retrospective chart-review study, it was not possible to ensure complete data were obtained for every child in the study (e.g., not every child had both frequencies/ears tested, and only a subset had actual BC-ABR thresholds). Threshold results for 500 Hz are particularly limited. Similarly, the timing and amount of data obtained at behavioural followup were variable. Without same day ABR and behavioural testing, changes in thresholds could not be controlled for. 

### 4.5. Clinical Implications

 The results of these studies have several key implications for clinical application. First, it is clear for both 2000 Hz and 500 Hz that normal/elevated cochlear sensitivity may be inferred from the presence/absence of a BC-ABR at 30 dBnHL for 2000 Hz and 20 dBnHL for 500 Hz. This is consistent with previous recommendations and current diagnostic protocols. 

When elevated, it is possible to categorize the degree of cochlear hearing loss, at least for 2000 Hz. For elevated 2000 Hz results, if no BC-ABR is present at 60 dBnHL then behavioural thresholds are greater than 65 dBHL. If BC-ABR is elevated but present between 35 and 60 dBnHL, then behavioural thresholds are 30–65 dBHL. If actual threshold is obtained, then the estimated BC behavioural threshold is equal to the BC-ABR threshold (i.e., a correction factor of 0 dB). This correction factor only applies when BC-ABR thresholds are elevated (i.e., does not apply if BC ABR is present at the normal level). For elevated 500 Hz results, Study B suggested that categorization is possible but limited ears with cochlear hearing loss preclude clinical application at this time. 

Future studies should investigate BC-ABR and behavioural thresholds obtained on the same day in infants with a wide range of sensorineural hearing loss, especially mild hearing loss. Additional threshold results at 500 Hz are particularly needed. Finally, as there are very few data in the literature for 1000 and/or 4000 Hz BC ABR, future studies should also investigate these frequencies in children with normal and impaired hearing.

## Figures and Tables

**Table 1:** Accuracy of “normal” BC-ABR levels for classification of “normal” versus “elevated” cochlear status.

			BC-ABR results	
			Normal	Elevated
2000 Hz	Behavioural outcome	Normal	28 (20.3%)	0 (0.0%)
138 ears		Elevated	3 (2.2%)	107 (77.5%)

500 Hz	Behavioural outcome	Normal	36 (61.0%)	0 (0.0%)
59 ears		Elevated	1 (1.7%)	22 (37.3%)

Percent of total ears in parentheses.

BC ABR “normal” levels: 30 and 20 dBnHL for 2000 and 500 Hz.

Data for the “certain” and “presumed SNHL” groups included.

**Table 2:** Comparison of BC-ABR category to AC behavioural threshold data for 2000 Hz and 500 Hz.

			BC-ABR category (dBnHL)		
			Normal	35–60	>60
2000 Hz 104 ears	Behavioural outcome category (dBHL)	Normal	28 (26.9%)	0 (0%)	0 (0%)
		30–65	1 (1.0%)	25 (24.0%)	3 (2.9%)
		>65	0 (0%)	1 (1.0%)	46 (44.2%)

			Normal	25–45	>50
500 Hz 47 ears	Behavioural outcome category (dBHL)	Normal	36 (76.6%)	0 (0%)	0 (0%)
		30–55	1 (2.1%)	5 (10.6%)	0 (0%)
		>55	0 (0%)	1 (2.1%)	4 (8.5%)

Percent of total ears in parentheses.

BC ABR “normal” levels: 30 and 20 dBnHL for 2000 and 500 Hz.

AC (and BC) behavioural “normal” levels are ≤25 dBHL.

Most behavioural threshold data are AC (2000 Hz: 89/104; 500 Hz: 33/47). See text for details.

**Table 3:** Comparison of BC-ABR category to BC behavioural threshold data for 2000 Hz and 500 Hz.

			BC-ABR category (dBnHL)		
			Normal	35–60	>60
2000 Hz 41 ears	Behavioural outcome category (dBHL)	Normal	9 (22.0%)	0 (0%)	0 (0%)
		30–65	1 (2.4%)	15 (36.6%)	1 (2.4%)
		>65	0 (0%)	0 (0%)	15 (36.6%)

			Normal	25–45	>50
500 Hz 19 ears	Behavioural outcome category (dBHL)	Normal	13 (68.4%)	0 (0%)	0 (0%)
		30–55	1 (5.3%)	1 (5.3%)	0 (0%)
		>55	0 (0%)	0 (0%)	4 (21.1%)

Percent of total ears in parentheses.

BC ABR “normal” levels: 30 and 20 dBnHL for 2000 and 500 Hz.

BC behavioural “normal” levels are ≤25 dBHL.
